# Mechanical Characterization of Thermoplastic Aligner Materials: Recommendations for Test Parameter Standardization

**DOI:** 10.1155/2019/8074827

**Published:** 2019-05-29

**Authors:** F. Elkholy, S. Schmidt, M. Amirkhani, F. Schmidt, B. G. Lapatki

**Affiliations:** ^1^Department of Orthodontics, University of Ulm, Ulm, Germany; ^2^Institute of Experimental Physics, University of Ulm, Ulm, Germany

## Abstract

**Background:**

Understanding of the different mechanical properties of thermoplastic materials is essential for a successful aligner treatment and further developments. However, data of previous material testing studies are scarcely comparable. Aim of the current study was to evaluate the different test parameters to lay the foundations for guidelines for future, more standardized three-point-bending aligner material tests.

**Materials and Methods:**

Several parameters concerning the specimen preparation and experimental three-point-bending setup were varied. The specimens were collected from polyethylene terephthalate glycol (PET-G) Duran® foils with different thicknesses. Both raw foils and foils thermoformed using different geometrical forms were investigated. The three-point-bending tests were performed using span lengths of 8 and 16 mm and variable deflection ranges between 0.1 and 0.2 mm. The influence of water storage on the bending forces was studied using unloaded and loaded specimens. Experimental results were validated using a beam cantilever mathematical model.

**Results:**

Local macroscopic cracks after long-term loading could be avoided by keeping the deflections within a thickness-dependent individual range. The mathematical calculations confirmed that these individual deflection ranges lead to local stresses between 14 and 18 MPa. Constantly loaded specimens immersed for 24 hours in water showed a decrease of the bending force by 50%. This reduction was much smaller for the unloaded specimens (14%).

**Conclusion:**

During clinical aligner therapy, very small bending deflections are combined with small distances between the tooth surface regions supporting the aligner. In vitro aligner material testing by three-point bending should consider these geometrical aspects, while keeping the material stresses in a range between 14 and 18 MPa to avoid local microcracks. Considering these aspects, thickness-dependent deflections were established for three-point bending of the PET-G specimen for a span length of 8 mm. We recommend the application of these test parameters in future aligner material studies to achieve valid and comparable test results.

## 1. Introduction

Aligners have gained great interest in the last decade as a substitute for a fixed orthodontic appliance. Aligner therapy is characterized by a stepwise correction of tooth malpositions. Each step is reflected by one setup model on which a thermoplastic appliance (i.e., the aligner) is fabricated. Principally, aligners force the teeth into the position predefined by the corresponding setup model which might be regarded as a “shape-driven” tooth movement approach.

Different materials and treatment concepts have been introduced since the introduction of aligners by Kesling in 1945 [1–5]. Currently, two main approaches are being clinically utilized. The first approach, e.g., represented by the Invisalign® system (Align Technology, Santa Clara, California, USA), is based on smaller setup increments ranging between 0.1 and 0.2 mm, while utilizing a single aligner with identical properties for each setup increment. The second approach, e.g., represented by the Clear Aligner system (Scheu Dental GmbH, Iserlohn, Germany), is based on larger setup increments (0.5–1.0 mm); within each of these increments, a sequence of aligners with increasing thickness in the range between 0.4 and 0.8 mm is utilized to compensate for the force decrease related to the tooth movement which makes larger setup increments possible.

Independent from the approach applied, tooth movement is induced by the discrepancy between the “programmed” tooth position in the setup model (and in its negative, i.e., the aligner) and the actual position of the patient's tooth. This discrepancy will induce local deformations of the aligner leading to the application of loads to the individual teeth which move the teeth into the desired position. The mechanical loading of the teeth is directly related to the mechanical properties of the material used. These properties are commonly tested in vitro by determination of load-deflection curves. In a more realistic setup, such tests may implement a model of a dental arch considering that tooth morphology and different directions of tooth movement influence the mechanical loads applied by the aligner on the individual teeth [6–16]. Other more simplified approaches principally correspond to either a “three-point-bending” or “single-axis tension” using untreated or thermoformed flat material samples of a defined size. The feasibility of the latter approaches is less complex, and results are better comparable since the test setup and material samples can be more easily standardized. This might explain the consideration of such approaches in ISO testing standards (ISO 20795-2 [17]).

Several studies have been published, reporting results from tensile tests or three-point-bending tests with different specimen geometries and dimensions [18–26]. Three-point-bending was mainly used to investigate clinical influencing factors such as thermocycling, water storage, and long-term loading on the material behavior in vitro. For instance, Kwon et al. examined the force delivery of different thermoplastic materials after thermocycling or repeated deflection in an experimental test apparatus with supports spanning 24 mm [22]. All tested specimens were cut from thermoplastic foils thermoformed on a flat stone model with the dimension 30 × 60 × 10 mm. Furthermore, all specimens were deflected to 1 mm independent of their thickness. It was concluded that the delivered forces are influenced by the material thickness and type. They also mentioned that the optimal forces for tipping a central incisor are achieved by a 0.5-mm polyethylene foil (Essix A+, Essix Raintree Co., New Orleans, LA, USA). This statement, however, is unsubstantiated as aligners thermoformed on jaw models will retain a three-dimensionally complex form corresponding to the dental surfaces. Such geometry leads to local reinforcement of the aligner and will, in turn, have a great influence on the forces delivered [6]. A further study carried by Iijima et al. also implemented three-point bending to characterize the mechanical properties of different untreated (i.e., not thermoformed) thermoplastic specimens during a temperature increase from 25°C to 100°C [25]. The supports of the three-point-bending setup spanned 12 mm, and deflections were 3 mm for all foil thicknesses. Results showed a distinct force decay of the specimen after 30 min with a reduction of 34% for the polyethylene terephthalate glycol (PET-G) Duran® (Scheu Dental GmbH, Iserlohn, Germany) material. Furthermore, the Duran® as well as two different experimental polyurethane (PU) materials showed relatively stable mechanical properties [25]. In a similar manner, Lombardo et al. evaluated the relaxation behavior of different thermoplastic materials during a 24-hour loading time [21]. The tests were performed in a three-point-bending setup with a span of 25 mm. The individual deflection value for each material/thickness was defined as one-fourth of the corresponding material's yield strength value [21]. Investigated specimens showed a higher stress relaxation during the first 8 hours after which a plateau was reached. The Duran® specimens revealed a stress relaxation value of 44% after the first 8 hours, reaching a value of 62% after a 24-hour loading period. It has to be noted, however, that in this study, the specimens were cut from raw blanks with no prior thermoforming. Furthermore, only the stress has been described for each material instead of the also reporting force magnitudes. Hence, results are scarcely comparable to those of previous studies. Examination of thermoplastic materials by three-point bending was also performed by Ryu et al. in order to test the changes in mechanical properties of thermoplastic materials after thermoforming [26]. In an attempt to simulate the thermoforming process on jaw models, the raw foils were thermoformed on a 40-mm-wide plastic mold with height and thickness simulating the (flattened) surface size of an upper central incisor [26]. Specimens collected after thermoforming indicated higher water absorption (determined by weight change) as well as a lower bending force compared to specimens collected from the raw foils.

Although these previous studies provide an important insight into different aligner materials, their results cannot be directly compared to each other due to the different experimental setups and procedures applied. Furthermore, little is known about the influence of the different experimental setup parameters on the study results. Hence, the aim of the present study series is to explore the influence of different experimental setting parameters on the results of thermoplastic material tests. These parameters include (1) the influence of the support distance during three-point-bending tests, (2) the form used for thermoforming, (3) the maximum deflection without appearance of cracking, and (4) the influence of water storage on the bending forces [18–26].

## 2. Materials and Methods

### 2.1. Tested Specimens and Preparation

**Figure 1:**
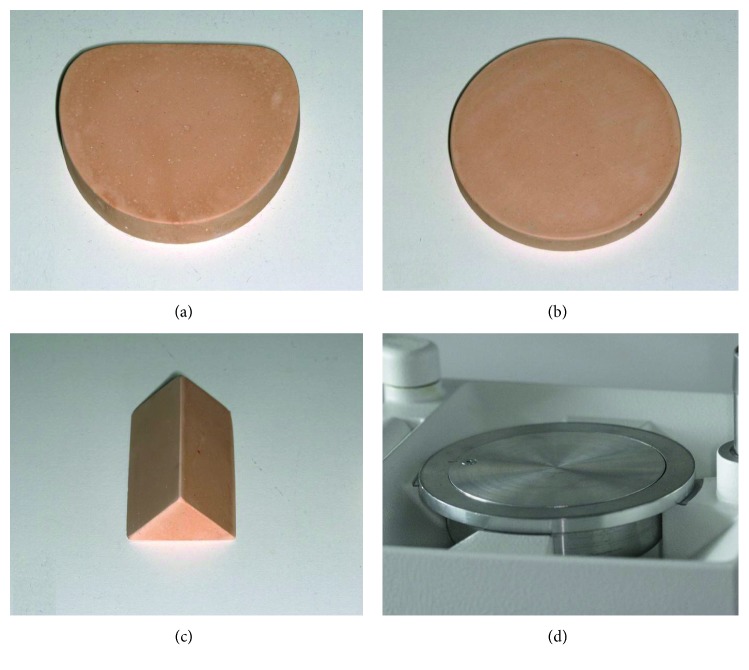
Different forms used for thermoforming the thermoplastic foils: (a) stone model base plate (*L* × *W* × *H* = 74 mm × 66 mm × 12 mm), (b) stone round disc (*H* = 9 mm, *r* = 35 mm), (c) gable roof shaped specimen (rise = 17 mm, span = 24 mm), and (d) stainless steel model holding plate.

PET-G foils (Duran®, Scheu Dental GmbH, Iserlohn, Germany) with thicknesses ranging from 0.4 mm to 0.75 mm were examined. Rectangular specimens 40 mm in length and 10 mm in width were collected from untreated (i.e., not thermoformed) as well as thermoformed foils. These specimens were extracted using a paper cutting guillotine (Dahle guillotine 517, Novus Dahle GmbH, Lingen, Germany) which avoided the occurrence of cutting burrs. For fabrication of thermoformed specimens, four different forms were utilized including a stainless steel model holding plate, a model base plate, a round disc, and gable roof shaped specimen (Figure 1). The latter three were fabricated with type IV dental stone. Independent from the specific form used, all tested specimens had the same dimension of 40 mm in width and 10 mm in length and were extracted from flat areas of the thermoformed foils. The thickness of all the tested specimens was measured in the middle portion using a digital micrometer gauge (Toolcraft B302-003, Georgensgmünd, Germany).

### 2.2. Three-Point-Bending Setup

**Figure 2:**
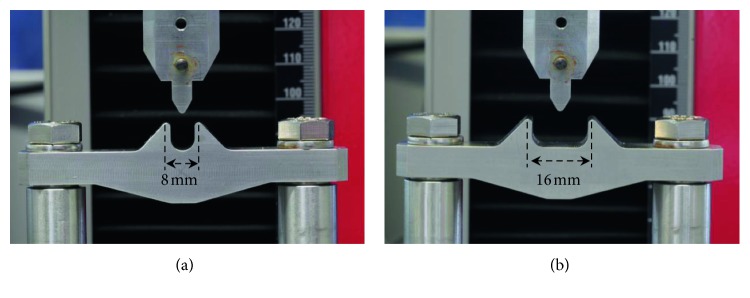
Detailed view of the three-point-bending test setup with the (a) 8-mm and (b) 16-mm span length.

A universal testing machine (Zwick Z2.5, Zwick-Roell, Ulm, Germany) equipped with a 100 N force sensor was used for three-point-bending of PET-G specimens (Figure 2). The test setup was constructed for measurements with distances between lateral supports of 8 mm and 16 mm, respectively (Figure 2). The two lateral supports and the cylindrical central support had a radius of 0.5 mm for both configurations. These components were made of stainless steel and were produced on a CNC milling machine. The setup was enclosed in a climate chamber to maintain a temperature of 37°C during the testing procedure. Additionally, the setup was equipped with a transparent acrylic basin to enable a complete immersion of the material specimen into double-distilled and tempered (37°C) water for some of the tests (see below).

### 2.3. Test Procedure

At begin of the experiments, the specimens were fixed in the universal testing machine, and the cylindrical central support was deflected to the predefined distance at a low speed of only 1 mm/min. Deflection forces were measured continuously throughout loading and unloading of the specimens.

In the first test series, untreated specimens collected from raw PET-G foils were bent using a lateral span length of either 8 mm or 16 mm. The central support was deflected by either 0.25 mm or 0.5 mm. Nine specimens were examined for each of the four configurations. For all further tests, specimens were extracted from thermoformed foils. For determination of the optimal individual deflection range for each specimen thickness, the specimens were initially deflected at 0.25 mm for 24 hours using only the 8-mm span length setup configuration. If any plastic deformation or cracks in the material were observed, the deflection was reduced for 0.05 mm, and a new specimen was tested using the new, reduced deflection range. This procedure was repeated until no plastic deformation or cracks occurred. The third test series aimed at examining the influence of water storage. Specimens were initially bent to the individualized maximum deflection. Subsequently, the specimens were immersed in 37°C distilled water for 24 hours without loading. Then, force delivery was again measured. In the fourth test series, specimens were initially bent to the individualized maximum deflection, before being immersed in 37°C distilled water for 24 hours. In contrast to the third test series, however, water storage occurred in the loaded state applying the individualized maximum deflection. Finally, delivered forces were measured again applying the individual maximum deflection range. Only force values for maximum deflections were included in the further analysis.

### 2.4. Mathematical Model

We used a mathematical model based on the Euler–Bernoulli beam theory to calculate different parameters within the linear elastic range and compare them to the measured values [27]. For all calculations, we considered an E-modulus of 2050 MPa as well as the original foil thicknesses, as given by the manufacturer. The first parameter considered was the effective force (*F*) for the different foil thicknesses with the 8-mm and 16-mm span length and was expressed according to equation (1), where *f* is the deflection range, *E* is the E-modulus, *I*_y_ is the second moment of area, and *l* is the span length. Additionally, the second moment of area was calculated using equation (2), where *b* is the specimen thickness and *h* is the width of the specimen, i.e., 10 mm. In the second part of the mathematical model, we used equation (3) with the previously mentioned parameters to calculate the maximum local stress (*σ*_max_) experienced by the different PET-G specimen thicknesses for deflection depths ranging between 0.05 and 0.3 mm.

(1) $$\begin{array}{c} F=\frac{48\cdot E\cdot I_{\text{y}}\cdot f\;}{l}, \end{array}$$

(2) $$\begin{array}{c} \;I_{\text{y}}=\frac{bh^{3}}{12}, \end{array}$$

(3) $$\begin{array}{c} \sigma_{\text{max}}=\frac{6\cdot f\cdot E\cdot h}{l^{2}}. \end{array}$$

## 3. Results

### 3.1. Influence of Span between Lateral Supports

**Figure 3:**
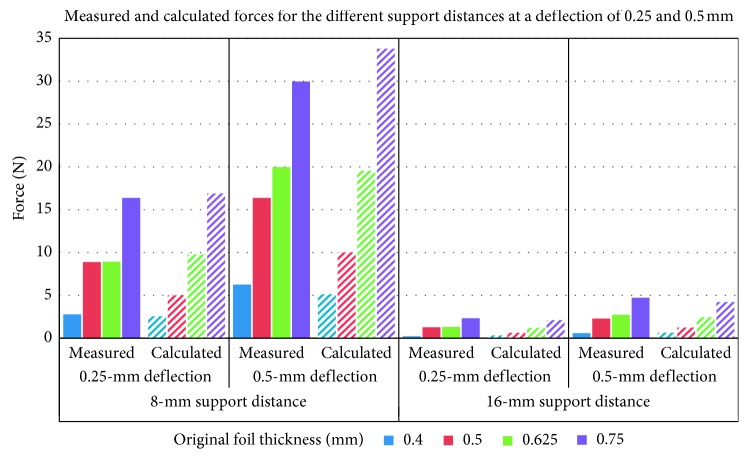
Measured (solid colours) and calculated (striped) maximum forces for dry, not thermoformed specimens. Values refer to different foil thicknesses for the 8 mm and 16 mm span lengths at 0.25 mm and 0.5 mm deflection depth.

The maximum forces measured for dry, not thermoformed PET-G specimens using support distances of 8 mm and 16 mm are represented in Table 1 and Figure 3. On average, forces measured during the 0.25-mm deflection were 51% of the forces measured during the 0.5-mm deflection. Values for the 16-mm span length reached on average only 13% of those for the 8-mm span length.

Similar forces with only slight differences were calculated using the mentioned mathematical model (equations (1) and (2)). The corresponding calculated values are presented in Table 2 and Figure 3.

### 3.2. Influence of Form Used for Thermoforming on Foil Thickness and Force Delivery

The thickness changes measured after thermoforming using the different forms are presented in Table 3. The highest thickness reduction was observed for the gable roof form (17%), followed by the stone model base plate (15%). Both the stone round disc and the metal model holding plate showed average thickness reductions of approximately 8%.

**Figure 4:**
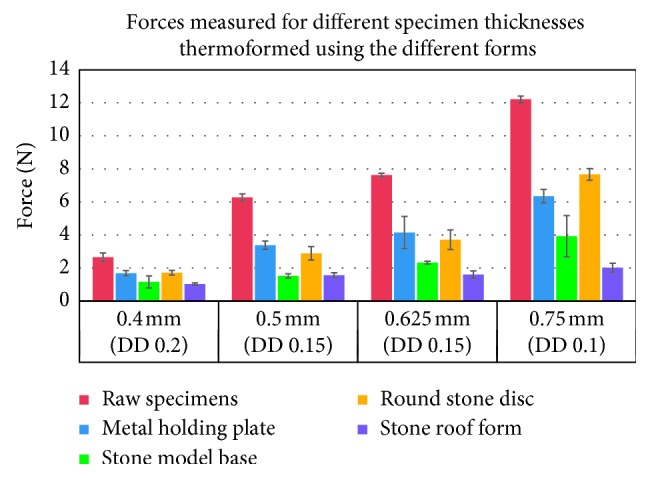
Maximum forces measured at the individually specified deflection depth (DD) for different PET-G specimen thicknesses. Specimens were thermoformed using the different forms. Forces are compared to those measured for the specimens extracted from the raw thermoplastic foils.

The forces measured before and after thermoforming using the different forms are presented in Table 4 and Figure 4. The individual maximal deflection range was used for each specimen thickness. The highest force reduction was observed for the gable roof form with an average value of 75%, followed by the model base form (67%). Similar to the thickness reduction, both the stone round disc and the metal model holding plate also showed, in average, similar reduction values of circa 44%. The metal model holding plate was used for thermoforming the foils included in all further tests.

### 3.3. Maximum Deflection without Appearance of Cracking

**Figure 5:**
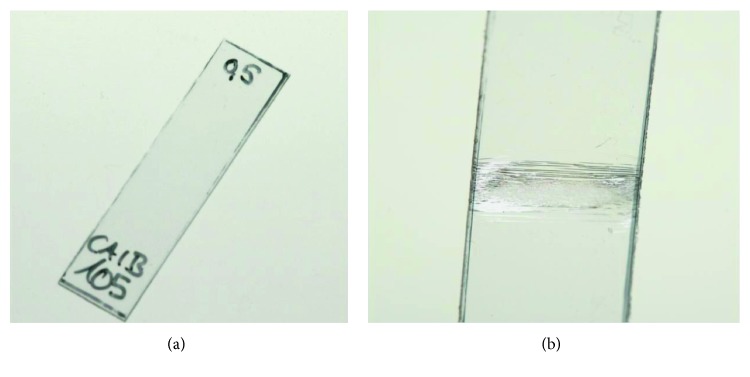
A 0.5-mm thick specimen with 40 mm length and 10 mm width (a) before and (b) after loading for 24 hours with a 0.5-mm deflection and 8-mm span length.

The effect of long-term loading is presented in Figure 5, showing a thermoformed 0.5-mm thick specimen before loading (Figure 5(a)) and after a 24-hour loading by a 0.5-mm deflection of the central support (Figure 5(b)). Cracks were visible in almost the whole specimen range between the 8-mm supports, with the highest concentration in the specimen's middle part. By contrast, when reducing the deflection range to 0.15 mm, both the 0.5-mm and 0.625-mm thick specimens showed no visible microfractures. The thickness-specific maximal deflection ranges which just did not lead to cracks or microfractures after 24-hour loading are given in Table 5. According to the applied mathematical model, the individually selected deflection depths in Table 5 induced local stresses ranging between 14.41 and 15.38 MPa for the 0.4-mm, 0.5-mm, and 0.75-mm specimens. For the 0.625-mm specimens, however, the calculated local stresses at a deflection of 0.15 mm were 18.02 MPa (Table 6).

### 3.4. Influence of Dry and Wet Specimen Storage with and without Specimen Loading on Force Delivery

**Figure 6:**
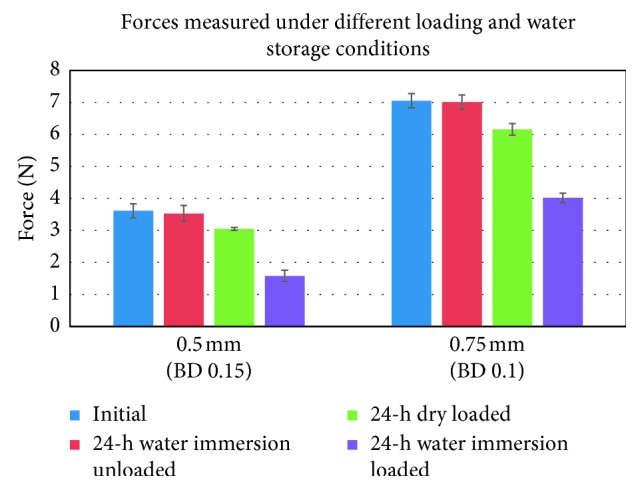
Forces measured for the 0.5-mm and 0.75-mm specimens before and after 24-hour water storage, as well as after long-term loading with and without water immersion. The error bars represent the standard deviation for the different conditions.

The forces measured at the individual deflection depths after water immersion of thermoformed specimens are presented in Table 7 and Figure 6. Both unloaded and loaded specimens were stored in water. Water immersion without loading resulted in a minimal force reduction of ca. 1% when compared to forces delivered by the same specimen before water storage. Constant loading of specimens for 24 hours in a dry environment led to a force reduction of circa 14%. Loading of the same specimen types combined with water storage, however, resulted in an average force reduction of ca. 50% when compared to the initial loading force of dry specimens.

## 4. Discussion

Biomechanical testing of aligners by three-point bending generally uses flat specimens. The fact that such approach does not consider the complex 3D morphology of aligners applied for clinical therapy has disadvantages and advantages. The main disadvantage lies in the fact that the obtained force and moment magnitudes have no direct clinical significance. By contrast, results of in vitro testing of aligners thermoformed on models of the full dental arches are relatively close to the clinical situation. The main advantage of three-point bending, on the other hand, is related to the simplified specimen geometry. Hence, construction of a test setup is relatively simple and inexpensive. Moreover, interpretation of the results in mechanical terms is relatively easy, because the underlying basic mechanics is well established and the number of influencing factors is limited. As a result, such simplified biomechanical testing particularly allows for the controlled evaluation of isolated factors influencing the force delivery of aligners.

Despite the simplified test approach, comparison of results of three-point-bending studies published in the literature is greatly limited due to the variability of the test setup parameters used [21]. One of the most influencing test parameters of three-point bending is the span length between the two lateral supports. Multiple testing standards were implemented and described in the literature, e.g., the ISO 20795-2 for base polymers and the ANSI/ADA Specification no. 32 for orthodontic wires [17, 21, 22, 25, 26]. These standards, however, focus on stiffer orthodontic materials which show a more even distribution of material stresses. The thermoplastic materials used in the aligner therapy, however, are clinically subjected to a higher stress concentration. To simulate these stresses, in combination with a span length of up to 32 mm as described in the previously mentioned testing standards, one would require much higher deflection ranges. In our experiments, we investigated the influence of two reduced span lengths on the bending forces. The first was the 16-mm span length in an attempt to simulate the width of two central incisors. The test results from this span length, however, should be interpreted cautiously as the tests were performed with flat specimens allowing a relatively even stress distribution over a larger area between both supports. The second span length applied was 8 mm. In our opinion, this span length would deliver more realistic results, due to the local stress concentration on smaller aligner areas during the clinical application with peak stresses at the proximal regions. This aspect would also explain the microcracks observed in the aligner after clinical application [28]. According to the results of the current study, changing the span length from 8 to 16 mm has led to an average force reduction of 86%. The mathematical model (equations (1) and (2)) resulted in similar bending forces, however, with slightly higher values (Tables 1 and 2). This difference was more visible with 0.5-mm foils and can be related to the “measured” thickness of the raw specimens with values reaching 0.56 mm (Table 3) instead of their nominal thickness of 0.5 mm. Nevertheless, the results indicate that the used mathematical model provides a good approximation for calculating the initial bending forces for different thermoplastic materials. This should be due to the nature of the performed measurements with very short loading periods. It seems that such loading time is not long enough for viscosity-related plastic deformation of the specimens and, consequently, a linear elastic material behavior is maintained.

On the other hand, increasing the deflection depth while keeping a constant span length showed a directly proportional effect on the deflection force. This effect was also confirmed by Kwon et al. [22]. As indicated by our results, an indicator for overloading the specimens would be the appearance of macroscopic cracks (Figure 5). This is usually due to overloading of the specimens beyond the recovery point, which leads to accelerating the failure of the bonds between the polymer chains. Therefore, this parameter should be varied depending on the material thickness allowing for nearly equal and, to a certain extent, comparable local stress for all specimen thicknesses (Table 6). An exception was the stresses calculated for the 0.625-mm specimens at a deflection range of 0.15 mm with values reaching 18.02 MPa. These specimens, however, also showed no visible cracks at this deflection range. This might be related to the thinning of the 0.625-mm PET-G foils after thermoforming to a thickness of about 0.6 mm inducing local stresses of circa 17.3 MPa (Table 3). In total, these results would suggest that maintaining a stress concentration below 14 MPa for the PET-G specimens would prevent a premature material integrity failure, independent from the thickness of the tested specimen. Further studies should, however, be performed to investigate the optimal stresses for different thermoplastic materials. It has to be noted that the given stresses are calculated from Young's modulus given by the manufacturer measured at room temperature according to the ISO 527. Hence, the stress values will slightly vary under the influence of temperature and humidity changes [29]. Nevertheless, the relationship between the calculated stresses for the different deflection depths in Table 6 will be the same.

Generally, thermoforming leads to thinning of the aligner material and, consequently, to a reduction of the forces delivered by the specimens [24, 26]. Such effect was also observed in the current study. It is quantitatively influenced by thermoforming variables such as the form used as well as the thermoforming temperature and pressure applied. With regard to the influence of the thermoforming pressure, we evaluated pilot measurements for specimens fabricated with a lower thermoforming pressure (4.8 bar) in contrast to the normally used pressure (5.9 bar). These pilot measurements, however, showed contradicting results; i.e., although the thickness of the specimens (measured in their center) is only changed marginally, the forces measured were higher for the specimens thermoformed by 4.8 bar device. Further studies with more specimens and multiple thickness measurement points along the span length are, however, needed for a more concise clarification of this phenomenon.

The influence of the thermoforming form is probably related to the topographical distribution of the thickness changes on the basic shape of the foil (i.e., the disk) and the location where the specimens are collected from the disk. Several attempts were made to simulate the thermoforming of thermoplastic foils on dentition models by using different geometrical forms resembling different teeth forms, e.g., the central incisors or simple stone blocks with different dimensions resembling a dentition cast [22, 24, 26]. Our results indicated that, independent from the form used, all thermoformed specimens showed a significant force reduction when compared to the specimens extracted from raw thermoplastic foils tested under the same conditions (Table 3). The highest force reduction of circa 75% was observed for the gable roof form. In comparison, the round disc and the metal holding plate showed lower average reductions of 44% for both forms. A general question is whether a complex geometrical form should be used for thermoforming in order to simulate the clinical process with a dentition model or not. In our opinion, three-point bending should use the most symmetric and simple form, because each attempt to simulate the complex 3D shape of a dental arch will fail anyway. Hence, we would recommend using a flat model holding plate as the best alternative form for thermoforming the foils for material testing. Such form leads to the most even thickness reduction of the thermoformed disk and, therefore, of the collected specimens. Such strategy consequently follows the concept of best possible standardization of three-point bending. Moreover, also a larger area would be available for specimen extraction from the thermoformed foils.

Another important influencing parameter for thermoplastic material testing is the influence of water storage which intends to simulate the intraoral environment. A remarkable finding of our tests was that 24-hour water storage without loading did not show a visible effect on the deflection forces measured (Table 7). By contrast, 24-hour water storage under constant load induced a 50% force reduction (compared to the 14% reduction observed during the 24-hour constant load in dry conditions). This result is likely to be related to the influence of the water storage on lowering the creep resistance of the PET-G material by reducing the mechanical cohesion and increasing the mobility of the polymer chains [30].

Taken together, the previously stated findings indicated the importance of the different specimen preparations and test parameters on the results of the mechanical testing of PET-G thermoplastic aligner materials and would aid in paving the path for more standardized study designs and comparable results. Although this study focused on PET-G thermoplastic materials, the findings may well be applied to other thermoplastic materials. The latter will be addressed in further studies.

According to the results of the current study, we would suggest applying the following parameters for standardizing three-point-bending tests of PET-G specimens:

- Tests should be performed using thermoformed specimens.
- A flat metal plate should be used during thermoforming.
- The span length (distance between the two lateral supports) should be 8 mm.
- The span length must ensure continuous support during the experiment. In the current study, we used specimens with a 40-mm length.
- The specimens should have a width of 10 mm.
- In addition to the tests with dry specimens, tests of specimens after water immersion should be performed under long-term loading with a minimum duration of 24 hours.
- Specimens fabricated from different materials and with different thicknesses should be tested using an individual deflection depth. This depth should ensure a constant maximum material stress just prior to the occurrence of microcracks (see equations (1)–(3) for interrelation between deflection depth and maximum stress). For PET-G, we would suggest a maximum stress value of 14 MPa.

## Figures and Tables

**Table 1:** Forces measured for dry, not thermoformed specimens at 0.25 mm and 0.5 mm deflections for the different specimen thicknesses with the 8 mm and 16 mm support distances.

Original foil thickness (mm)	Lateral support distance of 8 mm				Lateral support distance of 16 mm			
	0.25 mm deflection		0.5 mm deflection		0.25 mm deflection		0.5 mm deflection	
	Mean force (N)	SD for force (N)	Mean force (N)	SD for force (N)	Mean force (N)	SD for force (N)	Force (N)	SD for force (N)
0.4	2.78	0.07	6.25	0.39	0.22	0.00	0.59	0.01
0.5	8.88	0.18	16.36	0.24	1.28	0.04	2.28	0.05
0.625	8.94	0.20	19.98	0.63	1.31	0.04	2.74	0.20
0.75	16.39	0.33	29.97	0.65	2.33	0.04	4.73	0.20

The SD values represent the standard deviation of the corresponding forces.

**Table 2:** Effective forces (F) calculated at the 0.25-mm and 0.5-mm deflection for the different specimen thicknesses from not thermoformed foils with the 8-mm and 16-mm support distance.

Original foil thickness (mm)	Lateral support distance of 8 mm		Lateral support distance of 16 mm	
	0.25 mm deflection	0.5 mm deflection	0.25 mm deflection	0.5 mm deflection
0.4	2.56	5.13	0.32	0.64
0.5	5.00	10.01	0.63	1.25
0.625	9.78	19.55	1.22	2.44
0.75	16.89	33.78	2.11	4.22

**Table 3:** Measured average, maximum (max.), and minimum (min.) thicknesses for thermoformed specimens (TF) using the different thermoforming forms. The original foil thickness represents the thickness given by the manufacturer, and the measured thickness represents the thickness measured by the micrometer gauge. Thickness reduction due to thermoforming is given as a percentage value.

Original foil thickness (mm)	Before thermoforming			TF with flat metal plate			TF with stone model base			TF with round stone disc			TF with stone roof form		
	Thickness (mm)	Max.	Min.	Thickness (mm)	Max.	Min.	Thickness (mm)	Max.	Min.	Thickness (mm)	Max.	Min.	Thickness (mm)	Max.	Min.
0.4	0.42	0.42	0.40	0.38	0.39	0.37	0.35	0.38	0.33	0.38	0.38	0.37	0.35	0.36	0.34
0.5	0.56	0.56	0.55	0.51	0.51	0.50	0.45	0.46	0.44	0.50	0.51	0.48	0.45	0.46	0.44
0.625	0.63	0.63	0.63	0.60	0.60	0.59	0.54	0.55	0.54	0.58	0.60	0.56	0.52	0.52	0.51
0.75	0.77	0.77	0.77	0.72	0.73	0.71	0.68	0.70	0.65	0.74	0.74	0.73	0.65	0.65	0.64
Average reduction				−8%			−15%			−8%			−17%		

**Table 4:** Measured forces and calculated stresses (MPa) at the individual deflection depths for thermoformed specimens (TF) using different thermoforming forms. The SD values represent the standard deviation of the corresponding forces. Force and stress reduction due to thermoforming are given as percentage values.

Original foil thickness (mm)	Deflection depth (mm)	Raw foil			TF with flat metal plate			TF with stone model base			TF with round stone disc			TF with stone roof form		
		Mean force (N)	SD	Stress (MPa)	Mean force (N)	SD	Stress (MPa)	Mean force (N)	SD	Stress (MPa)	Mean force (N)	SD	Stress (MPa)	Mean force (N)	SD	Stress (MPa)
0.4	0.2	2.66	0.27	16.14	1.69	0.16	14.61	1.16	0.36	13.45	1.71	0.14	14.61	1.03	0.07	13.45
0.5	0.15	6.27	0.21	16.14	3.38	0.25	14.70	1.53	0.13	12.97	2.89	0.41	14.41	1.57	0.15	12.97
0.625	0.15	7.63	0.11	18.16	4.15	0.97	17.30	2.33	0.08	15.57	3.72	0.60	16.72	1.61	0.22	14.99
0.75	0.10	12.22	0.19	14.80	6.35	0.41	14.03	3.93	1.25	13.45	7.67	0.35	14.41	2.02	0.27	12.49
Reduction					−44%			−67%			−44%			−75%		

**Table 5:** The deflection ranges defined for the different specimen thicknesses without showing signs of material fatigue.

Original foil thickness (mm)	Deflection depth (mm)
0.4	0.20
0.5	0.15
0.625	0.15
0.75	0.10

**Table 6:** Calculated maximal local stresses (MPa) for the different foil thicknesses at different deflections with the 8-mm span length. The calculations were based on the foil thickness reported by the manufacturer. The numbers in italics represent the stresses for the experimentally tested deflection ranges.

Original foil thickness (mm)	Deflection depth (mm)					
	0.05 mm	0.1 mm	0.15 mm	0.2 mm	0.25 mm	0.3 mm
	Maximum stress (*σ*_max_) (MPa)					
0.4	3.84	7.69	11.53	*15.38*	19.22	23.06
0.5	4.80	9.61	*14.41*	19.22	24.02	28.83
0.625	6.01	12.01	*18.02*	24.02	30.03	36.04
0.75	7.21	*14.41*	21.62	28.83	36.04	43.24

**Table 7:** Forces measured for the 0.5-mm and 0.75-mm thick specimens before and after water storage, as well as the forces measured with specimens of the same thicknesses after a 24-hour constant load with and without water storage. All specimens were loaded to the predetermined deflection of 0.15 mm and 0.10 mm for the 0.5-mm and 0.75-mm specimens, respectively. The SD values represent the standard deviation of the corresponding forces.

Original foil thickness (mm)	Deflection depth (mm)	Initial forces		After 24-hour water storage without load		24-hour constant load in dry condition		24-hour constant load in water	
		Mean force (N)	SD	Mean force (N)	SD	Mean force (N)	SD	Mean force (N)	SD
0.5	0.15	3.61	0.22	3.05	0.05	3.53	0.25	1.58	0.18
0.75	0.10	7.05	0.22	6.16	0.18	7.01	0.22	4.02	0.14
Average reduction				−1%		−14%		−50%	

## Data Availability

The data (images and tables) used to support the findings of this study are included within the article.
